# What can we learn about joint degeneration from rare and orphan diseases?

**DOI:** 10.1186/1471-2474-16-S1-S17

**Published:** 2015-12-01

**Authors:** JA Gallagher

**Affiliations:** 1 Musculoskeletal Biology, IACD, University of Liverpool, Liverpool, UK

## 

William Harvey, the great English physician of the 17th century, observed *“Nature is nowhere accustomed more openly to display her secret mysteries than in cases where she shows tracings of her workings apart from the beaten paths; nor is there any better way to advance the proper practice of medicine than to give our minds to the discovery of the usual law of nature, by careful investigation of cases of rarer forms of disease”*[1]. The history of medical science has proven Harvey correct; studying severe phenotypes of rare diseases has helped elucidate pathophysiological mechanisms of more common disorders and led to the identification of new biomarkers and therapeutic targets [2]. For example the development of bisphosphonates, the most successful class of bone active agent, owes a debt to research on hypophosphatasia. More recent research on rare bone syndromes has helped identify new targets to inhibit bone resorption and stimulate bone formation including cathepsin K and sclerostin. Drugs against both these targets are now in clinical trials. Osteoarthritis (OA) is a major cause of morbidity and disability. It is also the only major musculoskeletal disorder for which there are no effective therapies, other than pain relief and eventual joint replacement. Recent studies on rare cartilage syndromes have identified some potential therapeutic target including GDF5 and lubricin.

Research from our laboratory has focussed on the early onset, aggressive joint destruction which occurs in the osteoarthropathy of the rare disease alkaptonuria (AKU). AKU is a single gene defect in tyrosine metabolism, which is characterised by ochronosis, the deposition of pigmented polymers in connective tissues particularly cartilage. Studying tissue samples from AKU patients and from AKU mouse models has revealed significant parallels with the pathophysiology of OA. We have discovered several previously unidentified microanatomical changes in AKU joints which were subsequently recognised in joint degeneration associated with OA and ageing. Of these the most significant are high density mineralised protrusions (HDMPs). These novel micoanatomical structures arise via the extrusion of a mineralisable matrix through cracks in the subchondral plate. Formation of HDMPS constitutes a previously unrecognised mechanism of joint destruction [3].

## References

[B1] Harvey W Letter IX, to John Vlackveld 1657

[B2] Gallagher JA Ranganath LR Boyde A Lessons from rare diseases of cartilage and bone Curr Opin Pharmacol 2015 22 107 114 25978274 10.1016/j.coph.2015.04.002

[B3] Boyde A Davis GR Mills D Zikmund T Cox TM Adams VL On fragmenting, densely mineralised acellular protrusions into articular cartilage and their possible role in osteoarthritis J Anat 2014 225 4 436 446 25132002 10.1111/joa.12226 PMC4174026

